# Recent insights into the microRNA-dependent modulation of gliomas from pathogenesis to diagnosis and treatment

**DOI:** 10.1186/s11658-022-00354-4

**Published:** 2022-08-03

**Authors:** Alireza Mafi, Atefe Rahmati, Zahra Babaei Aghdam, Raziyeh Salami, Marziyeh Salami, Omid Vakili, Esmat Aghadavod

**Affiliations:** 1 Department of Clinical Biochemistry, School of Pharmacy and Pharmaceutical Sciences, Isfahan University of Medical Sciences, Isfahan, Iran; 2 Department of Hematology and Blood Banking, Faculty of Medicine, Mashhad University of Medical Sciences, Mashhad, Iran; 3 Department of Basic Science, Neyshabur University of Medical Science, Neyshabur, Iran; 4 Imaging Sciences Research Group, Tabriz University of Medical Sciences, Tabriz, Iran; 5 Department of Clinical Biochemistry, School of Medicine, Hamadan University of Medical Sciences, Hamadan, Iran; 6 Department of Clinical Biochemistry, School of Medicine, Shahid Sadoughi University of Medical Sciences, Yazd, Iran; 7 Research Center for Biochemistry and Nutrition in Metabolic Diseases, Kashan University of Medical Sciences, Kashan, Iran; 8 Department of Clinical Biochemistry, School of Medicine, Kashan University of Medical Sciences, Kashan, Iran

**Keywords:** Glioma, Brain neoplasms, MicroRNAs, Carcinogenesis, Biomarkers, Therapeutics

## Abstract

Gliomas are the most lethal primary brain tumors in adults. These highly invasive tumors have poor 5-year survival for patients. Gliomas are principally characterized by rapid diffusion as well as high levels of cellular heterogeneity. However, to date, the exact pathogenic mechanisms, contributing to gliomas remain ambiguous. MicroRNAs (miRNAs), as small noncoding RNAs of about 20 nucleotides in length, are known as chief modulators of different biological processes at both transcriptional and posttranscriptional levels. More recently, it has been revealed that these noncoding RNA molecules have essential roles in tumorigenesis and progression of multiple cancers, including gliomas. Interestingly, miRNAs are able to modulate diverse cancer-related processes such as cell proliferation and apoptosis, invasion and migration, differentiation and stemness, angiogenesis, and drug resistance; thus, impaired miRNAs may result in deterioration of gliomas. Additionally, miRNAs can be secreted into cerebrospinal fluid (CSF), as well as the bloodstream, and transported between normal and tumor cells freely or by exosomes, converting them into potential diagnostic and/or prognostic biomarkers for gliomas. They would also be great therapeutic agents, especially if they could cross the blood–brain barrier (BBB). Accordingly, in the current review, the contribution of miRNAs to glioma pathogenesis is first discussed, then their glioma-related diagnostic/prognostic and therapeutic potential is highlighted briefly.

## Background

Noncoding RNAs (ncRNAs) are a group of RNA molecules that either have a limited ability to encode a protein or lack it. Currently, ncRNAs are classified into two major groups of long noncoding RNAs (> 200 nts) and small noncoding RNAs (18–200 nts), according to their length. The family of small noncoding RNAs consists of small nucleolar RNAs (snoRNAs), Piwi-interacting RNAs (piRNAs), small nuclear RNAs (snRNAs), tRNA-derived small RNA (tsRNA), and microRNAs (miRNAs). NcRNAs were previously thought to be genomic dark matter, but studies have shown that these structures make up about 60% of the human genome [1]. To date, numerous evaluations have also revealed that ncRNAs involve in regulating the physiological and pathological processes of many human diseases, including multiple cancers [2].

miRNAs, as noncoding, small endogenous RNA molecules with an average length of 20–22 nucleotides, are widely distributed and highly conserved, with cell- and tissue-specific expression patterns. These RNA molecules principally contribute to the regulation of transcription, translation, or epigenetic modification of various genes [3–5]. The discovery of the first miRNA, *lin-4*, in *Caenorhabditis elegans* by the research teams of Ambros and Ruvkun was a milestone in molecular biology. Since then, several investigations have focused on this topic, providing evidence for the involvement of different miRNAs in biological processes, such as cell proliferation, cellular differentiation, apoptosis, and inflammation [2, 6]. miRNAs can posttranscriptionally regulate gene expression by binding to specific sites, acting as miRNA response elements (MREs), in their target transcripts, which result in transcript eradication or translation suppression. More interestingly, the expression levels of miRNAs may be deregulated in multiple cancers, converting them into oncogenes or tumor suppressors.

miRNAs have been reported to be associated with the formation and development of gliomas [7]. Gliomas are known to be the most lethal and common malignant brain tumors, having poor prognosis. Unfortunately, the 5-year survival for patients diagnosed with glioblastoma is only 10%. Through affecting the onset and progression of gliomas, miRNAs regulate the expression of particular genes involved in cancer-related processes. Considering the expression profile of miRNAs in glioblastoma, it has been proposed that miRNAs can impact genes, regulating cell proliferation, invasion, angiogenesis, apoptosis, and even chemoresistance. Furthermore, several miRNAs have been shown to exhibit tumor-suppressive effects in glioblastoma pathophysiology. Surprisingly, miRNAs have also been considered to be suitable tools for identifying the origin of gliomas, as well as improving the prognosis of patients and monitoring their response to treatment. Moreover, these ncRNAs have also been suggested to function as appropriate targets for glioma therapy [8, 9].

In this context, the current review aims to discuss the substantial relationship between particular miRNAs and the pathogenesis and progression of gliomas, with a special focus on their roles in the diagnosis and treatment of glial tumors. Although a significant number of reviews have highlighted the role of miRNAs in the pathogenesis of gliomas, while others have discussed the therapeutic/diagnostic significance of miRNAs, no recently published review has focused on both the pathogenesis and diagnostic/therapeutic aspects. To the best of the authors’ knowledge, this review thus provides an update that focuses on the role(s) of miRNAs in gliomas, from pathogenesis to diagnosis and treatment. To achieve the objective, the most recent publications in the field are reviewed and fully covered (Table 1).

**Table 1:** MicroRNAs involved in the pathogenesis of glioma

miRNA	Expression pattern	Study model	Sample type	Malignant behavior	Target(s)	Ref.
miR-21	Upregulated	In vitro In vivo	U87 glioma cell lines Xenograft tumor	Inhibition of apoptosis	Destabilizing TNFRs and decreasing the molecules involved in apoptosis including caspase-3, caspase-9, and APAF1	[93]
miR-221/222	Upregulated	In vitro In vivo	U251 glioblastoma/rat C6 glioma cells Xenograft tumor model (U251 glioma)	Induce proliferation of cell and disrupt cell death	Cell cycle inhibitors including p27 and/or p57	[96]
miR-34a	Downregulated	In vitro	MSCs-U87 U87-MG glioblastoma	Inhibition of glioma cell growth and cell cycle progression	Direct inhibition of expression of various cellular factors such as c-MET RTK, Notch-1, Notch-2, CDK6, CCND1, and SIRT1	[99, 100]
miR-124-3p	Downregulated	*Human* In vitro	Tissue samples U251 and U87 glioma cell lines/SHG44 glioblastoma cell line	Disrupt glioma cell proliferation, invasion, and migration	Connection with a variety of extracellular receptors such as NRP-1	[106, 107]
miR-125b	Upregulated	In vitro	U343 and U251 glioma cells	Induction of glioma cell proliferation, as well as inhibiting ATRA-induced cell apoptosis	Interaction with the 3′-UTR of Bmf	[108]
miR-9	Downregulated	*Human* In vitro	Tissue samples U87 MG (CL-0238) and TG-905 (CL-0309) glioblastoma cell lines	Inhibition of glioma cells proliferation and increased apoptosis	FOXG1 signaling	[122]
miR-186	Downregulated	*Human* In vitro	Tissue samples U87 glioma/astrocyte HA1800 cell lines	Increasing apoptosis	Inhibition of Smad6	[123]
miR-142-3p	Downregulated	*Human* In vitro	Tissue samples U251 and U87 cell lines	Inhibition of glioma cells proliferation and mitigates apoptosis	Inactivated Wnt/β-catenin signaling and activated caspase-3 signaling by targeting HMGB1	[124]
miR-25	Upregulated	*Human* In vitro	Tissue samples U-373MG Uppsala, U-87MG Uppsala, U251MG, and T98G glioma cell lines	Induction of tumor cell migration, invasiveness, and proliferation	Increasing expression of CADM2	[133]
miR-146b-5p	Downregulated	*Human* In vitro	Tissue samples U87MG/SNB19 cell lines	Inhibition of glioma cells migration and invasion	Degradation of MMP16-related mRNA	[139]
miR-379-5p	Downregulated	In vitro	U87 and U251 glioma cell lines/human astrocyte normal cells/HEB cells	Inhibition of glioma cells viability, migration, invasion, and EMT	Modulation of MGST1	[140]
miR-665	Downregulated	*Human* In vitro	Tissue samples U251MG, A172, LN18, and T98G glioma cell lines	Inhibition of glioma cell proliferation, migration, and invasion	Targeting HMGB1, as well as inhibiting the Wnt/β-catenin pathway	[143]
miR-3175 miR-134	Upregulated Downregulated	*Human* In vitro	Tissue samples U251 glioma cells	Affecting cell proliferation and apoptosis, as well as EMT	PI3K/Akt pathway	[148, 149]
miR-296	Downregulated	*Human* In vitro	Tissue samples U251 glioma cell line	Inhibition of cell invasion and multidrug resistance	Particular types of potassium channels such as EAG1 (also called KCNH1)	[150]
miR-320a	Downregulated	*Human* In vitro	Tissue samples U87 and U251 human glioma cell lines	Impeding invasion and migration of glioma cells	Aquaporin 4	[151]
miR-125b	Downregulated	*Human* In vitro	Pediatric low-grade glioma-derived cell lines (Res186, Res259, and BT66)	A decrease in cell growth and induction of apoptosis	The mechanisms is not clear	[155]
miR-125b	Downregulated	In vitro	Human brain microvascular endothelial cells	Inhibition of tumor angiogenesis	Molecular loop, which interacts with MAZ, VEGF, and miR-125b	[155]
miR-210-3P	Upregulated	In vitro	U87-MG, A172, and HS683 glioma cell lines	Induction of invasiveness and EMT in glioma cells Probable involvement in regulation of mitochondrial function	Modification of the mitochondrial membrane potential to improve mitochondrial function	[102]
miR-93	Upregulated	*Human*	Tissue samples	Induction of proliferation, cell cycle progression, colony formation, migration, invasion, and chemoresistance in glioma cells	Integrin β8	[158]
miR-124-3p	Downregulated	*Human* In vitro	Tissue samples U87MG and U251MG cell lines	Inhibition of the proliferation, invasion, and migration of glioma cells	Activating the PI3K/Akt/NF-κB signaling pathway	[152]
miR-21	Upregulated	In vitro	U87MG glioma cell line	Inhibition of TMZ-induced apoptosis	Decreasing the Bax/Bcl-2 ratio and caspase-3 activity	[167]
miR-125b-2	Upregulated	*Human* In vitro	Primary culture of glioblastoma tissues-derived cells	Induction of resistance of human glioblastoma stem cells to TMZ	Mitochondrial pathway of apoptosis	[169]
miR-128 and miR-149	Downregulated	*Human* In vitro	Tissue samples U251 and U87 glioma cell line	Inhibition of invasion of glioblastoma cells Increasing the TMZ sensitivity of glioblastoma cells	Rap1B-mediated cytoskeletal and related molecular alterations	[171]
miR-181a/b/c/d	Downregulated	*Human* In vitro	Tissue samples U251 and U87 glioma cell line	Increasing the TMZ sensitivity of glioblastoma cells Inhibition of invasive proliferation of glioblastoma cells	Rap1B-mediated cytoskeleton remodeling and related molecular (Cdc42, RhoA, and N-cadherin) changes	[172]
miR-155-3p	Upregulated	*Human* In vitro In vivo	Tissue samples LN299, A172, T98, U87, and U251 glioma cell lines and healthy human astrocytes Xenograft mice model (U87)	Induction of cell growth, while its inhibition promotes TMZ sensitivity Induction of cell arrest at the same point that TMZ acts on G1/S phase	Upmodulation of Six1 at translational level	[175]
miR-141-3p	Upregulated	*Human* In vitro In vivo	Tissue samples A172, U87, U251, T98, and LN229 glioma cell lines Xenograft mice model (U87)	Induction of cell growth and drug resistance Induction of tumor growth, as well as inhibition of cell apoptosis and cell cycle arrest	Suppression of p53 and its downstream proteins such as CDK2 and cyclin E1/B1, thus inhibiting cell cycle arrest at G1 to S phases	[176]
miR-195	Downregulated	In vitro	U251MG glioma cell line	Inhibition of miR-195-induced TMZ resistance and suppression of cell apoptosis of glioma cells	CCNE1	[177]
miR-524-5p miR-324-5p	Downregulated	*Human* In vitro In vivo	Profiling upon 158 glioma samples U87 and U251 glioma cells Xenograft mice model (U87)	Upregulation of miR-524-5p and miR-324-5p reduces glioma cell proliferation, but increases TMZ sensitivity	EZH2	[180]
miR-137	Downregulated	*Human*	Tissue samples	Inhibition of GSC self-renewal, while promoting their differentiation	Targeting RTVP-1, which downregulates CXCR4	[188]
miR-26a	Upregulated	In vitro In vivo	U251, U87, A172, and SHG44 glioma cell lines, human cervical cancer cell line HeLa, human embryonic kidney 293 and 293 T cells Xenograft mice model (U87)	Decreasing AP-2α expression by binding to the 3′-UTR of AP-2α and reversed the tumor-suppressive role of AP-2α in glioma	AP-2α/Nanog signaling axis	[192]
miR-30a	Downregulated	*Human* In vitro In vivo	Tissue samples A172, U87, and U251 glioma cell lines Xenograft mice model	Suppression of self-renewal and tumorigenicity of GSCs Exerting some antitumor effects on GSCs	Blocking the NT5E-dependent Akt signaling pathway	[194]
miR-30c	–	In vitro	Rat C6 glioma cell line	C6 cell sphere formation and neural differentiation to astrocytes	JAK–STAT signaling pathway	[195]
miR-33a	Upregulated	*Human* In vivo	Tissue samples Xenograft mice model	Increasing the PDE8A and UVRAG mRNAs’ expression	Modulation of PKA and Notch endocytosis signaling pathways	[196]
miR-300	Upregulated	*Human* In vivo	Tissue samples Xenograft mice model	Increasing the cell proliferation and self-renewal-related activities in patient-derived GSCs	Direct suppression of LZTS2	[239]
miR-29a	Downregulated	*Human* In vitro In vivo	Tissue samples U87 and U251 glioma cell lines BALB/c nude mice	Suppression of proliferation, migration, and invasion, but promotion of glioma cell apoptosis	Aberrated expression of MDM2/4 and final deregulation of the activity of the p53-miR-29a-MDM2/4 feedback loop	[173]

*Akt* protein kinase B; *APAF1* apoptotic protease activating factor 1; *ATRA* all-*trans* retinoic acid; *Bax* Bcl-2-associated X; *Bcl-2* B cell lymphoma protein -2; *Bmf* Bcl-2 modifying factor; *CADM2* cell adhesion molecule 2; *CCND1* Cyclin D1; *Cdc42* cell division cycle 42; *CDK* cyclin-dependent kinase; *CXCR4* C-X-C chemokine receptor type 4; *EAG1* Ether-à-go-go1 potassium channel; *EMT* epithelial–mesenchymal transition; *EZH2* Enhancer of zeste 2 polycomb repressive complex 2 subunit; *FOXG1* Forkhead box G1; *HMGB1* high mobility group box 1; *KCNH1* potassium voltage-gated channel subfamily H member 1; *LZTS2* leucine-zipper tumor suppressor 2; *MAZ* MYC associated zinc finger protein; *MDM2* mouse double minute 2; *MGST1* microsomal glutathione S-transferase 1; *MMP16* matrix metalloproteinase 16; *MSCs* mesenchymal stem cells; *NF-κB* nuclear factor-κB; *NRP* neuropilin; *NT5E* 5′-nucleotidase ecto; *PDE8A* phosphodiesterase 8A; *PI3K* phosphoinositide 3-kinase; *Rap1B* Ras-associated protein 1B; *RhoA* Ras homolog family member A; *RTK* receptor tyrosine kinase; *SIRT1* Sirtuin1; *Six1* sine oculis homeobox 1; *TMZ* temozolomide; *TNFRs* tumor necrosis factor receptors; *UTR* untranslated region; *UVRAG* UV radiation resistance-associated gene; *VEGF* vascular endothelial growth factor

## Gliomas

Gliomas are referred to as primary central nervous system (CNS) (esp. brain) tumors, resulting in substantial degrees of morbidity and mortality owing to their origin and locally invasive growth. These brain tumors represent approximately 30% of all primary CNS tumors, and 80% of all malignant ones. Based on their histological and immunobiological characteristics, gliomas can be categorized into astrocytomas, brain stem gliomas, ependymomas, mixed gliomas (so-called oligoastrocytomas), oligodendrogliomas, and glioblastomas [10]. In 2016, the updated version of the World Health Organization (WHO) grading system classified brain tumors principally based on the absence or presence of anaplastic features into four different grades from I to IV, including nuclear atypia, mitotic activity, microvascular proliferation, and/or necrosis. However, gliomas are classified as either low-grade (grades I or II) or high-grade (grades III and IV) tumors, depending on their growth potential and invasiveness [11].

Astrocytomas are slow-growing tumors that originate from astrocytes. These tumors can be either circumscribed, pilocytic astrocytomas, or infiltrative, diffuse astrocytomas. Histologically, pilocytic astrocytomas exhibit low to moderate cellularity and reduced mitotic activity, whereas diffuse tumors are characterized by a moderate increase in cellularity, mild to moderate nuclear atypia, and low degrees of mitotic activity. Oligodendrogliomas are generally low-grade, slow-growing tumors that develop from oligodendrocytes. Nevertheless, grade III oligodendrogliomas, which are also called anaplastic oligodendrogliomas, are considered to be malignant, fast-growing tumors [12]. Low-grade oligodendrogliomas have a relatively low proliferative index and well-delineated borders, while high-grade anaplastic oligodendrogliomas exhibit increased cell proliferation, nuclear atypia, and an altered rate of mitotic activity. On the other hand, tumors originating from ependymal cells can be classified into three distinct subgroups based on the WHO classification; grade I, including sub-ependymomas and myxopapillary; grade II, as slow-growing benign tumors; grade III, as invasive malignant cancer cells [13].

The most common gliomas in adults are the most infiltrative ones, including diffuse astrocytomas (WHO grade II), anaplastic astrocytomas (WHO grade III), glioblastomas (WHO grade IV), oligodendrogliomas (WHO grade II), and the controversial group of mixed oligoastrocytomas. Other tumors, such as pilocytic astrocytomas, pleomorphic xanthoastrocytomas, and ependymomas, are less common and have more favorable prognosis. In children, gliomas most commonly present in the form of pilocytic astrocytomas and diffuse midline gliomas in multiple grades, including diffuse intrinsic pontine gliomas [11, 13]. Nevertheless, respective studies of CNS tumors, such as ependymomas, have reported that patients cannot be reliably classified by employing only histological classifications, meaning that tumors with similar histological grades may result in substantially different survival outcomes. The fifth edition of the WHO classification of CNS tumors in 2021 employed a histo-molecular-based approach with confirmed prognostic and therapeutic advantages [14]. Indeed, the newly edited guideline in 2021 enhanced the previous classification system by employing novel molecular diagnostics, such as DNA methylome profiling [14].

Glioblastoma multiforme (GBM), the most common and mortal subtype of glioma, even after routine therapeutic methods, including resection surgery, followed by radiochemotherapy, has an average survival of approximately 6–12 months from detection [15, 16]. GBM is commonly categorized into two different clinical subtypes: primary and secondary. Approximately 95% of GBMs are primary, typically occurring de novo within a period of 3–6 months in the elderly. Meanwhile, secondary GBMs usually develop in younger patients with positive history of low-grade astrocytoma. Although the primary and secondary subtypes differ at the molecular level, their outcomes are almost identical because the same pathways are usually affected, thus the response to treatment also remains the same [15].

In the case of glioma therapy, which is guided by the WHO classification system, surgery is generally required, by either complete resection or a biopsy [15]. However, the treatment of patients with gliomas consists of a combination of resection, radiotherapy, and chemotherapy [17]. The goal of tumor resection is to remove all tumor tissue while minimizing the neurological risk to the patient. In this context, surgical resection has been reported to be more beneficial for glioblastoma than for WHO grade II and III gliomas [17]. The resection of recurrent glioblastoma cannot be beneficial alone and should be performed as part of an overall therapeutic approach. Since most recurrences of glioma arise in the immediate vicinity of the tumor, local radiotherapy of the tumor region or its bed can be employed following surgical resection [16, 18, 19]. In most cases, chemotherapy is also needed to maximize the efficiency of oncological treatment. The standard chemotherapeutic agent for gliomas is temozolomide (TMZ), while a combination of procarbazine, lomustine, and vincristine can be used for primary chemotherapy, as well as the treatment of recurrent gliomas [20, 21]. Although neurooncological studies are currently working on immunotherapeutic strategies and several trials have focused on these therapeutic approaches, no significantly positive outcomes have been obtained yet [17].

In the case of glioma detection, the final diagnosis is obtained by direct tissue assessment of glial tumors through surgical biopsies, which has inevitable risks. Thus, noninvasive strategies for the identification of brain tumors have attracted great attention to limit biopsy-related risks [22]. Although the BBB presents some challenges to accessing genetic material, some molecules have been reported to be secreted from tumor cells into the peripheral blood and the CSF of patients with glioblastoma [23]. miRNAs are perfect molecules in this context, with the ability to be secreted from tumor cells within extracellular vesicles [8, 24]. These ncRNAs are also reported to have particular regulatory roles in the tumor microenvironment [8, 22]. Therefore, miRNAs could be considered as potential diagnostic means, as well as promising therapeutic targets for the management of gliomas. The following sections provide much information about miRNAs and their functions in physiological as well as cancerous conditions.

## miRNAs

miRNAs are endogenous small single-stranded ncRNAs that are about 17–25 nucleotides long, involved in gene modulation, as well as epigenetic modifications. Given their prominent roles in many biological events, the potential of miRNAs as novel diagnostic, prognostic, and therapeutic targets has been constantly studied [25–27]. More than 50% of the genes responsible for the biogenesis of miRNAs are located at fragile chromosomal sites or cancer-associated genomic regions, suggesting key roles for miRNAs in the development of human malignancies [28]. Moreover, miRNA dysregulation is implicated in the progression of cancers by disrupting the expression of tumor suppressors and/or oncogenes [28, 29].

**Fig. 1:**
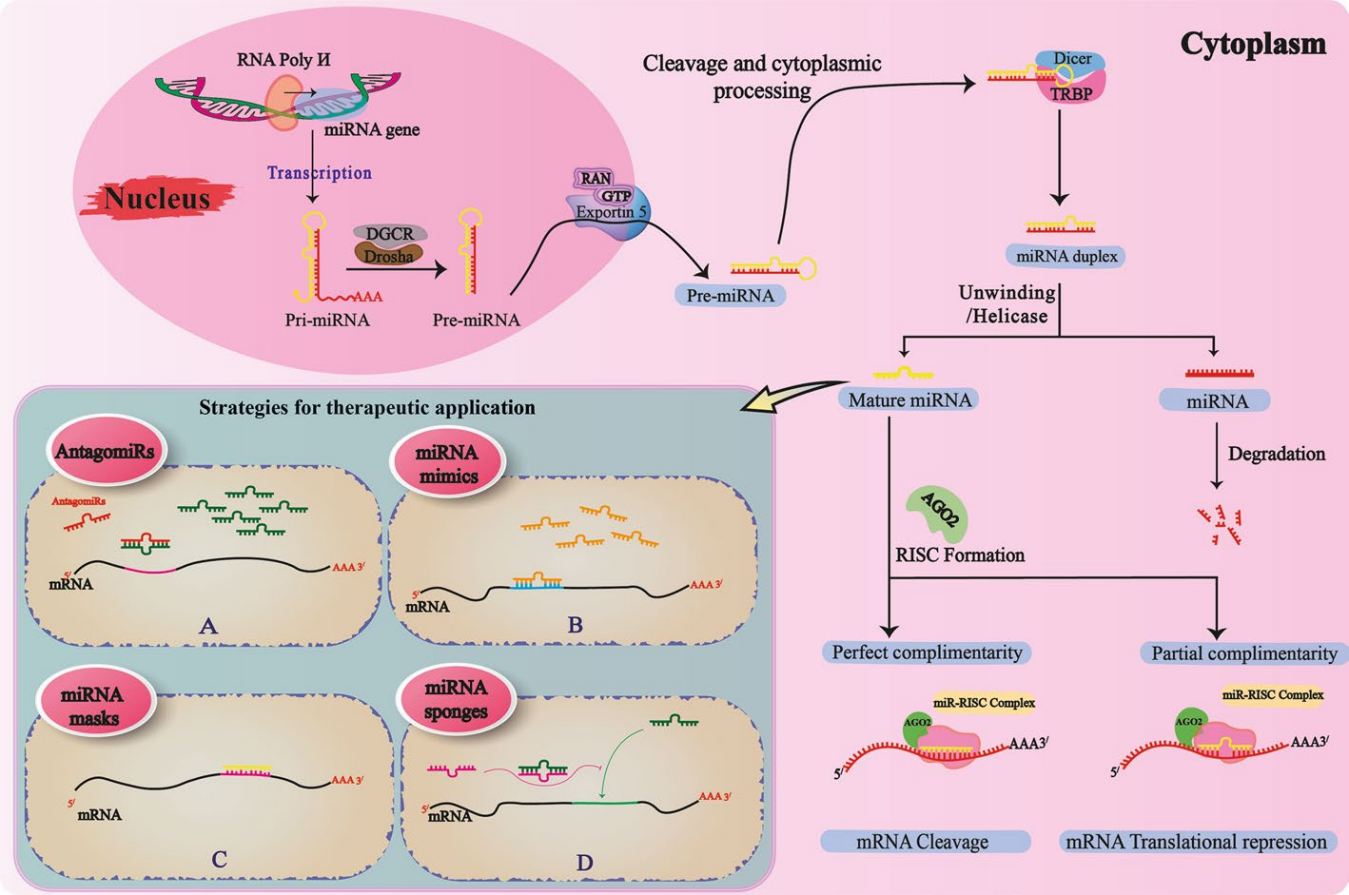
Processing of miRNAs, from biogenesis to therapeutic strategies. miRNAs are transcribed from particular genes inside the nucleus through the action of RNA polymerase II. After the formation of pri-miRNA, this is developed to pre-miRNA by the Drosha–DGCR complex. Then, the pre-miRNA leaves the nucleus for the cytoplasm via exportin 5. Following particular cytoplasmic processing and the action of the Dicer–TRBP complex, pre-miRNA is converted into duplex miRNA, which then undergoes an unwinding to produce mature miRNA. Mature miRNA, using the Ago-2 protein, forms a complex with RISC to cleave the mRNA of interest, or suppresses the translation process. In the case of therapeutic strategies attributed to miRNAs, there are four distinct strategies: **A** *AntagomiRs* that bind to and inhibit the action of oncomiRs by blocking miRNA-to-mRNA attachment, through a process called antisense action. AntagomiRs are also responsible for further degradation of miRNAs; **B** *miRNA mimics*, which help anticarcinogenic miRNAs to induce tumor-suppressive activities by reversing the epigenetic silencing; **C** *miRNA masks* that prevent miRNAs from acting on mRNAs by masking the 3′-UTR sequence on the mRNA strand; **D** *miRNA sponges*, whose behavior prevents miRNAs from acting on mRNAs by occupying the binding sites of a particular miRNA or even a set of miRNAs with similar seed sequences by a complementary RNA sequence. *Ago2* Argonaute RISC catalytic component 2;* DGCR* DiGeorge syndrome critical region;* GTP* guanosine triphosphate;* RISC* RNA-induced silencing complex;* RNA poly* RNA polymerase;* TRBP* TAR RNA binding protein

Molecular analyses have demonstrated that almost 50% of all human miRNAs are intragenic, originated from introns, while only a few derive from exons of protein-coding transcripts. The rest of miRNAs are generated from intergenic noncoding precursor-miRNA transcripts that are strictly regulated by their promoters [28, 30]. Note that miRNAs can regulate gene expression levels not only through translational repression but also by degradation of target mRNAs [31]. The biogenesis of miRNAs is a process that takes place in both the nucleus and the cytoplasm [32]. The initial step of transcription of miRNA genes in the nucleus is mostly done by RNA polymerase II to primary transcripts (pri-miRNAs) that are polyadenylated and have a cap [33, 34]. The pri-miRNAs generated may have several hairpin-like structures. These structures are then processed into characteristic stem-loop precursor miRNAs that are approximately 70 nucleotides in length and then modified by Drosha ribonuclease III in the microprocessor complex and its binding partner DGCR8 to the pre-miRNAs. Pre-miRNAs produced by the Ran-GTP/exportin 5 complex leave the nucleus for the cytoplasm [35]. In the cytoplasm, the structure of the Pre-miRNAs stem loop is further cleaved by the other RNase III enzyme, Dicer, and its cofactors, TAR RNA-binding protein (TRBP), resulting in duplex miRNAs of approximately 22 base pairs (bp). One strand of miRNA is rapidly degraded, while the other strand (the guide strand) is loaded into the RNA-induced silencing complex [36]. The RISC is a multiprotein assemblage of the Argonaute [37] family of proteins that along with mature miRNA, is essential for miRNA-mediated gene silencing. The generated miRNA–RISC complex binds to the target 3′-untranslated region (UTR) mRNA by identifying specific binding sites. Then, depending on the degree of complementarity of the linkages between miRNA and the target mRNA, effects such as translation inhibition or cleavage of mRNA will occur [38–40] (Fig. 1).

Specifically, miRNA-mediated gene expression control is crucial for the cellular response to environmental stresses, thereby being implicated in human diseases such as cancers. Hence, the expression of miRNAs may be altered as disease progresses. Furthermore, among all small ncRNAs, miRNAs are the most well-studied RNA molecules as promising biomarkers for diseases such as cancers, aging, and neurodegenerative disorders [41].

## miRNAs in cancer

Several studies have shown that miRNAs play fundamental roles not only in the normal functions of cells but also in the pathogenesis of malignant diseases. Indeed, miRNAs can behave as tumor suppressor or oncogene molecules via regulation of other protein-coding genes [28].

So far, multiple large-scale studies have demonstrated that miRNA profiling of cancers may be beneficial for staging, prognosis, and monitoring the therapeutic response. Microarray investigations on human cancers, cell lines, and nonmalignant cells have revealed 217 miRNAs involved in the tumorigenesis of multiple human malignancies [42]. Mounting evidence also indicates an important role for miRNAs in the development of cancers including breast, prostate, lung, pancreatic, and colon cancers, retinoblastoma, and glioblastoma [43]. The mechanisms of miRNA dysregulation in these malignancies include chromosomal abnormalities, amplification or deletion of miRNAs, transcriptional regulator alterations, epigenetic modifications, and alterations in the miRNA biogenesis machinery [28, 43]. Deletion or downregulation of two miRNA genes, miR-15a/16-1, which frequently occurs in patients with chronic lymphocytic leukemia (CLL), was the first evidence of miRNA involvement in human cancers [44].

Additional studies on lung cancer have reported that underexpression of miR-143 and miR-145 occurs as a result of deletion of the 5q33 region [45]. In contrast, the gene cluster miR-17–92 is amplified in lung cancers and B-cell lymphomas, as well as T-cell acute lymphoblastic leukemia [46, 47]. Overexpression of these miRNAs has been suggested to result from miRNA cluster gene translocation.

Since miRNAs are strictly regulated by different transcription factors, altered expression of miRNAs in cancer may be caused by dysregulation of some transcription factors, such as c-Myc and p53 [48]. The proto-oncogene *c-Myc* plays a fundamental regulatory role in growth control, cell proliferation, differentiation, and apoptosis, and alterations in its expression are associated with many tumors. A growing body of evidence has shown that there is a close interaction between c-Myc and miRNAs, with c-Myc being one of the main regulators of miRNA expression; transcripts of a number of tumor suppressor miRNAs such as miR-15a/16-1, miR-26, miR-29, miR-30, miR-34a, and the let-7 family are repressed by c-Myc [49, 50]. Another example of the regulatory role of c-Myc is reported in aggressive B-cell lymphoma (BCL) and acute myeloid leukemia (AML), where c-Myc is implicated in the repression of miR-26a. Conversely, in GBM, c-Myc upregulates miR-26a expression, promoting tumor cell proliferation [51].

The *p53* tumor suppressor continues to be known as the most frequently mutated gene in human cancers. Extensive literature has revealed a close relationship between p53 and miRNAs, with p53 regulating miRNAs at the transcriptional level and promoting the processing/maturation of a group of miRNAs (or certain miRNAs) [52]. Members of the miR-34 family were the first reported and the most prevalent p53-induced miRNAs, in this context [53]. Both 1p36.22 and 11q23.1 chromosome genes, which encode members of the miR-34 family, harbor several p53-responsive elements to which p53 can attach to activate transcription [53]. In human cancers, epigenetic alterations include DNA methylation, histone or chromatin posttranscriptional modifications (PTM), miRNAs, and nucleosome remodeling (or regulation) [54]. Interestingly, miR-127 levels were dramatically increased in cancer cell lines after treatment with DNA methylation, and histone deacetylase inhibitors [55].

## Gene mutations and signaling pathways contributing to glioma

In-depth understanding of the molecular characteristics of cancer, genetic and epigenetic abnormalities, and disturbances in signal transduction pathways can provide new insights into the identification and application of molecular biomarkers, as well as the design and development of potential chemotherapeutic approaches [50].

The generally accepted theory is that cancers are principally the result of progressive genetic and epigenetic alterations involved in cell proliferation and homeostasis [56]. Similarly, the pathogenesis of gliomas is also characterized by the sequential accumulation of genetic changes and abnormal regulation of growth factor signaling pathways that eventually lead to malignant transformation [57, 58]. Low-grade gliomas are commonly presented by two genetic abnormalities: (I) mutations in the TP53 tumor suppressor gene (associated with astrocytomas), and concurrent deletion of chromosomes 1p and 19q, seen in oligodendrogliomas [59]. On the other hand, high-grade gliomas exhibit altered expression of any of the p16INK4a, cyclin-dependent kinase 4 (*CDK4*), or retinoblastoma 1 (*RB1*) genes, resulting in the loss of normal *RB1* function [60, 61].

Mutations in isocitrate dehydrogenase (*IDH*), vascular endothelial growth factor (*VEGF*), epidermal growth factor (*EGF*), platelet-derived growth factor (*PDGF*), and hepatocyte growth factor (*HGF*) have been recognized to be involved in aberrant proliferation of glioma cells [62]. In general, genes encoding IDH enzymes are commonly mutated in various types of human cancer, including gliomas. Indeed, according to the WHO classification, diffuse astrocytic and oligodendroglial tumors are classified based on *IDH* mutations. Additionally, *IDH* mutations are frequently seen in secondary GBM [14, 62].

There are three different isoforms of IDH, which mediate multiple primary cellular metabolic functions; IDH1 is mainly found in the cytoplasm and peroxisomes, while IDH2 and IDH3 are located within the inner mitochondrial membrane [63]. Acquisition of mutant IDH1/2 would result in incomplete reprogramming of cellular metabolism [63]. IDH-mutant enzymes exhibit low affinity for isocitrate, thus preventing the formation of a-ketoglutarate (a-KG). However, these mutations also cause an increased binding affinity for nicotinamide adenine dinucleotide phosphate (NADPH), leading to a considerable increase in cytoplasmic levels of 2-hydroxyglutarate (2-HG), which is an oncometabolite, responsible for tumorigenesis. Indeed, 2-HG itself unloads carbohydrates from the citric acid cycle, making other non-Krebs-cycle sources of metabolites such as glutamine, glutamate, and branched-chain amino acids (BCAA) serve as alternative sources to support cellular metabolism [64, 65]. These findings have substantial implications for cancer therapy, as *IDH* inhibitors successfully use 2-HG suppression to hinder *IDH*-mutant glioma cells, which are more sensitive to glutaminase inhibitors [64].

The oncometabolite 2-HG not only changes the tumor metabolism but also involves in epigenetic alterations. In primary gliomas, high levels of 2-HG are associated with increased histone methylation and induction of differentiation [66]. Hence, by altering the status of histone and DNA methylation, IDH mutations alter gene expression patterns, leading to the inhibition of progenitor cell differentiation and the promotion of tumorigenesis along with subsequent oncogenic mutations [66, 67]. In addition, primary GBMs usually exhibit amplification of mouse double minute 2 (*MDM2*), which is a negative regulator of the tumor suppressor p53, phosphate and tensin homolog (*PTEN*) mutations, and homozygous deletion involving the *CDKN2A* tumor suppressor gene, whereas secondary GBMs are more likely to exhibit *p53* mutations, *IDH* mutations, *MET* amplification, and overexpression of *PDGFRA* [68].

Signal transduction is a multistep process by which a cell responds to an extracellular stimulus using intracellular signaling molecules, initiated via binding of an extracellular messenger to a cell surface receptor. The signal transduction pathways highly regulate cell proliferation, differentiation, the cell cycle, and cell death, thus playing a central role in the search for molecular targets that could be exploited for therapeutic purposes [69]. Therefore, signal transduction regulates the cellular process via transmembrane receptor kinase, which is activated by growth factors, cytokines, and hormones. These cellular receptors include epidermal growth factor receptor (EGFR), vascular endothelial growth factor receptor (VEGFR), platelet-derived growth factor receptor (PDGFR), insulin-like growth factor 1 receptor (IGF-1R), and fibroblast growth factor receptor (FGFR), which all are receptor tyrosine kinases (RTKs) [70]. RTKs are a family of cell surface receptors that can regulate cell proliferation, differentiation, and metabolic pathways as well as the cell cycle [69, 70].

Three main signaling pathways involve in gliomas pathogenesis, including RB1 (retinoblastoma 1 gene), p53 pathway, and signaling mediated by RTK/Ras/phosphoinositide 3-kinase (PI3K) and downstream molecules [71]. Molecular mutations, turning protooncogenes into oncogenes can overactivate these signaling pathways, whereas inactivation of tumor suppressors would remove these crucial negative regulators [72].

As mentioned above, aberrations in the components of the RB pathway such as CDK4/6, RB, CCND, INK4A, and E2Fs have been implicated in gliomagenesis or transformation/progression from low- to high-grade astrocytoma [73]. The tumor suppressor Rb plays a critical role in regulating the cell cycle through its interaction with the transcription factors of the E2F family and various chromatin modifiers and remodelers with a contributing role in cell cycle progression [74]. Once Rb binds to E2F proteins, it negatively regulates the G1-to-S phase transition by suppressing E2F target genes such as *CCNA1* and *CCNE1*. Conversely, in response to growth stimuli (or mitogen stimulation), the cyclin-dependent kinases (CDK4/6 and CDK2) phosphorylate Rb, releasing E2F, which allows the use of transcription factors and facilitates G1-to-S phase progression [74, 75]. However, as revealed by the Cancer Genome Atlas (TCGA) project, about 80% of primary GBMs exhibit abnormalities in this pathway, including *RB1*, *CDKN2A* gene deletion or mutation, and/or *CDK4 *gene amplification [76].

The most frequently altered gene is TP53, associated with human cancers. A deregulated p53/ARF/Mdm2 pathway has been proved to be central to GBM cell proliferation, invasion and migration, apoptosis evasion, and stemness [77]. In unstressed cells, p53 activity is low and negatively regulated through constant ubiquitination and proteasomal degradation by Mdm2/MdmX [78]. In response to stress, the interaction between p53 and Mdm2 is lost, leading to p53 accumulation, which further induces cell cycle arrest and/or apoptosis [78, 79]. According to the TCGA report, p53/ARF/Mdm2 is deregulated in nearly 84% of GBMs, which results in diminished tumor suppressor activity [76]. The most common mutations in the *p53* gene are considered to be missense mutations, leading to the overexpression of oncogenic variants of p53 protein and homozygous deletions of *CDKN2A/ARF*, which cause degradation of p53 and/or amplification of *Mdm2* and *Mdm4*, further resulting in loss of p53’s various tumor-suppressive activities [80].

Collectively, GBMs principally exhibit amplification and/or mutations of RTKs, either the EGFRs or the PDGFRAs. The amplification of the *EGFR* gene that encodes an altered EGFR protein occurs mostly in primary GBMs, whereas secondary GBMs exhibit dysregulation of PDGFR signaling. As the result of both mutations, the activity of multiple signaling pathways downstream to these RTKs, such as PI3K/Akt/rapamycin-sensitive mTOR complex (mTOR) pathways, is increased [81]. The PI3K/Akt/mTOR pathway is critical to many aspects of normal cellular functions as well as pathologic conditions such as oncogenesis and cancer progression [82]. In GBMs, overactivation of this pathway is found to be correlated with poor patient survival and tumor aggressiveness as it overstimulates processes responsible for cell proliferation, survival, and migration in malignant glial cells [83].

Epigenetic modifications are also present in different types of cancer, including gliomas. Mutations in epigenetic regulator genes are now known to drive certain types of glioma phenotype [84]. These changes include *IDH1* or *IDH2* mutations in low-grade gliomas, recurrent somatic heterozygous mutations in the gene encoding the histone variant H3 in high-grade childhood gliomas that are connected with DNA methylation profile [56, 85].

## miRNAs and gliomas

During the last few decades, much effort has been invested in identifying miRNAs that have abnormal expression patterns in gliomas and selecting the most promising ones to be evaluated for use in therapeutic strategies. Several studies have identified various miRNAs with potential functions in gliomas [86]. One of the earliest and most extensively studied cancer-promoting “onco-miRNAs” is miRNA-21. Interestingly, it was the only one to be found to be increased in all types of solid cancers, as well as the first to be found to be deregulated in human glioblastoma [37]. Its overexpression is linked to tumor growth by impairing apoptosis in malignant glial cells [87]. To date, multiple evaluations have focused on the miRNA signature in glioma tissue samples versus noncancerous brain tissues, to identify the role of miRNAs in the glioma pathogenesis in an effort to illustrate the underlying mechanisms [86].

**Fig. 2:**
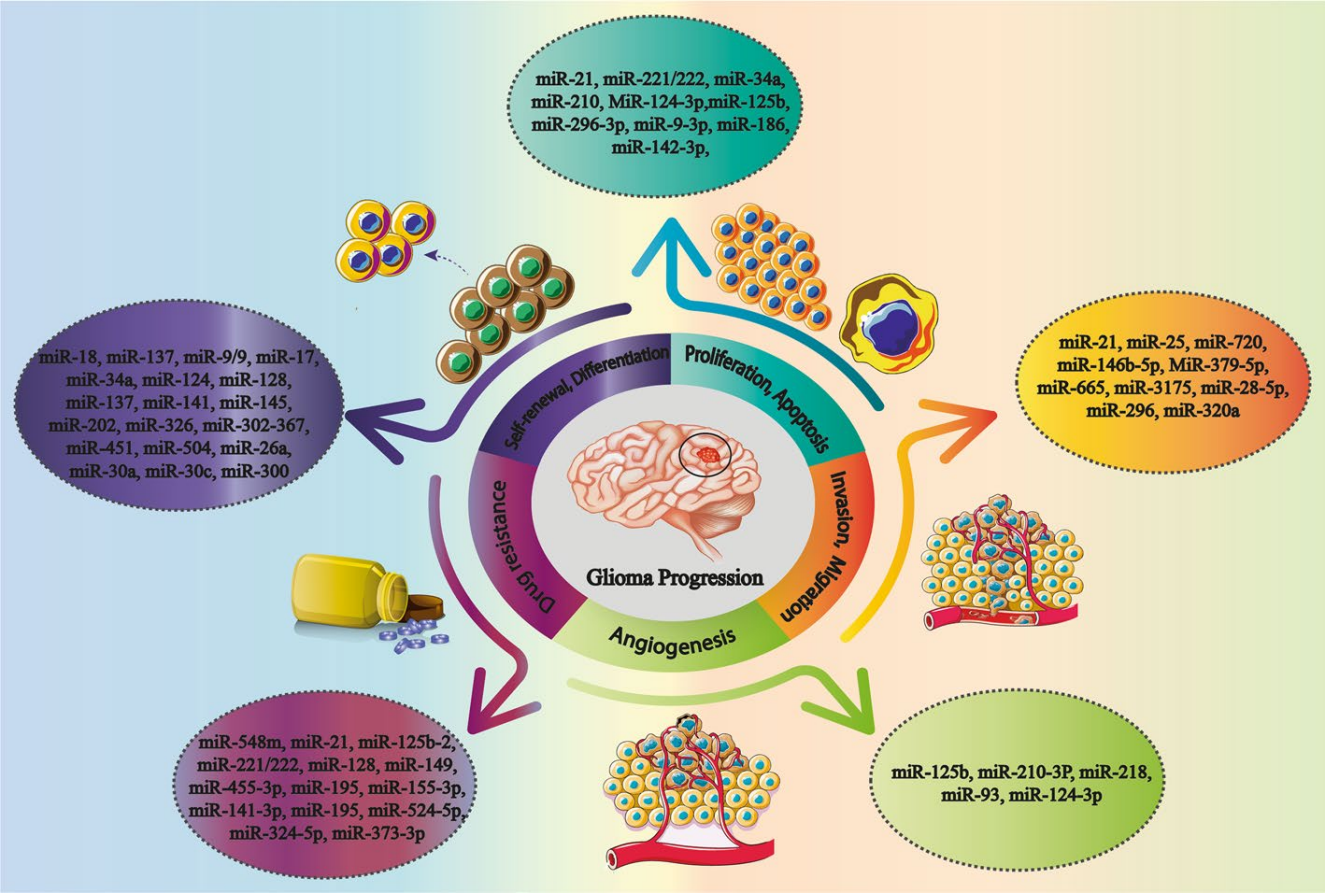
Schematic view of the relationship between miRNAs and the glioma progression

The expression profiles of miRNAs in tumor margin and non-tumor brain tissue have been found to be significantly different, as 256 miRNAs were significantly upregulated in GBM tissues compared with normal brains. These miRNAs include miR-21, members of the miR-17/92 cluster (miR-17, miR-18a, miR-19a, miR-19b-1, miR-20a, and miR-92a-1), miR-93, miR-221, and miR-222, with the ability to modulate several hallmarks of cancer, including cell proliferation, angiogenesis, invasion, metastasis, and drug resistance, while 95 miRNAs including miR-7, miR-34a, miR-128, miR-137, miR-181, etc. were downregulated in GBM, where their over expression might inhibit GBM development [88]. In the context of the glioma–miRNA relationship, the current review generally focuses on the contribution of miRNAs to the pathogenesis and progression of gliomas, as highlighted in the following sections (Fig. 2).

### miRNAs involved in cell proliferation and apoptosis

The continuous cell proliferation ability in cancer represents an imbalance between cellular proliferation and apoptosis, which increases as the grade of differentiation decreases [89]. As mentioned above, one of the first miRNAs found to be deregulated in glioma was miR-21, which is extremely upregulated in glioma and its expression levels are strongly associated with tumor grade and prognosis [90]. A variety of processes are modulated by miR-21, and studies have determined that the regulation of this miRNA promotes glial cell proliferation [90–92]. In greater detail, miR-21 exerts its antiapoptotic effects by destabilizing TNF-α receptors (TNFRs) on the cell surface by deregulation of the TIMP3–TNFR interaction (the extrinsic pathway of apoptosis), and/or decreasing caspase-3, caspase-9, and cytosolic apoptotic peptidase activating factor 1 (APAF1) (intrinsic-mitochondrial pathway), or even induction of lower caspase-3/7 activity as key components of all cellular apoptotic pathways [93].

miR-21 targets several tumor suppressor genes known to be critical regulators of apoptosis or cell proliferation, including programmed cell death 4 (PDCD4) [92], acidic nuclear phosphoprotein 32 family member A (ANP32A) [91], PTEN [90], and Sprouty 2 (SPRY2) [94]. Additionally, miR-21 can also direct several components of p53, TGF-β pathways, namely TAp63 (tumor suppressor homolog of p53), p53 activating cofactors and homologs, heterogeneous nuclear ribonucleoprotein K (HNPRK), key TGF-β factors such as TGF-β receptors, and proapoptotic death-domain-associated protein Daxx, leading to promotion of glioma cell proliferation and inhibition of apoptosis [95].

miR-221 and miR-222, which are defined as gene clusters (miR-221/222), are notably upmodulated in many human disorders, including cancers. In glioma, miR-221/222 act as oncomiRs and target the cell cycle inhibitors p27 and/or p57, thus regulating cell cycle progression from the G1 to S phase [96]. In addition, miR-221/222 can promote cell proliferation and inhibit cell death by targeting of p53-upregulated modulator of apoptosis (PUMA) which is a proapoptotic protein. Under physiological conditions, PUMA binds to Bcl-2-like proteins such as Bcl-2, B-cell lymphoma-extra-large (Bcl-xL), and myeloid cell leukemia sequence 1 (Mcl-1), thereby freeing Bax and/or Bak, which are then able to trigger apoptosis through mitochondrial dysfunction and caspase activation [97].

On the other hand, miR-34a is a tumor-suppressive miRNA with low expression levels in various human cancers, and also is the first miRNA demonstrated to be directly regulated by the tumor suppressor p53 [98]. In glioma, miR-34a suppresses cell cycle progression and proliferation of glioma cells through direct inhibition of the expression of MET receptor tyrosine kinases (c-MET RTK), Notch-1, Notch-2, cyclin-dependent kinase 6 (CDK6), cyclin 1 (CCND1), and silent information regulator 1 (SIRT1) [99, 100].

The other miRNA that mediates cell proliferation and apoptosis in GBM is miR-210 [101]. miR-210 is a particular target of hypoxia-inducible factor-1 (HIF-1), which is overexpressed as an adaptive response to hypoxic conditions within a tumor [102]. Bcl-2 19 kD interacting protein (BNIP3), one of the enhancers of cell apoptosis, was identified as a direct functional target of miR-210 [103]. When BNIP3 gene expression is induced, it localizes to mitochondria, resulting in a loss of membrane integrity, oxidative stress, mitochondrial damage, and eventually cell death. Through direct inhibition of BNIP3 expression, miR-210 reduces cell death [103, 104]. Furthermore, regulator of differentiation 1 (ROD1) is a miR-210 target, being involved in GBM progression. MiR-210 inhibits the proliferation of tumor cells and induces the apoptotic flux by negative regulation of ROD1 [105].

MiR-124-3p is also recognized as a mediator of the cell cycle and survival/apoptosis in glioma cells. Flow cytometric analyses of cancer tissues and relevant cell lines have shown overexpression of miR-124-3p. Surprisingly, this miRNA has been verified to be a negative regulator of Neuropilin-1 (NRP-1), which is a multifunctional receptor involved in glioma cell proliferation, invasion, and migration through binding to various extracellular receptors [106, 107].

Xia et al. demonstrated a positive relationship between overexpression of miR-125b and glioma cell proliferation, as well as suppressed apoptosis [108]. This brain-enriched miRNA was reported to be distributed among neurons and astrocytes [109]. Prior to this exploration, miR-125b overexpression had been detected in oligodendroglial tumors and brain specimens of fetuses with Down syndrome [110, 111]. In vitro evaluations have indicated that miR-125b depletion would repress the proliferation of human neuroblastoma cells [112]. In the context of the roles of miR-125b in provoking glioma cell proliferation and inhibiting all-*trans*-retinoic acid (ATRA)-induced cell apoptosis, underexpression of miR-125b sensitizes cells to ATRA-induced apoptosis. Furthermore, negative crosstalk was found between miR-125b and the apoptosis-related protein Bcl-2 modifying factor (BMF), in which miR-125b can link to 3′-untranslated region (UTR) of BMF [108].

ICAT, which is defined as the inhibitor of β-catenin and T cell factor (TCF), is a perfect negative regulator of Wnt signaling, functioning by blocking the TCF-to-β-catenin attachment [113]. Although ICAT has been found to be deregulated in a group of human malignancies, its carcinogenic functions remain undetermined [59]. In GBM, ICAT downmodulation has been reported to inhibit cell proliferation, migration, and invasion, while stimulate the apoptotic flux [114]. More interestingly, the expression of ICAT can be modulated by different miRNAs in various different types of cancer, including hepatocellular carcinoma (HCC), breast cancer, and GBM [115, 116]. On the other hand, miR-296-3p is also downmodulated in GBM tissues compared to normal brain [117].

The overexpression of miR-296-3p has been shown to be linked to lower survival rates according to the Cancer Genome Atlas GBM dataset [118]. Surprisingly, the expression of ICAT has been revealed to be inversely associated with miR-296-3p expression in GBM tissues. Consistently, ICAT is underexpressed in grade II gliomas, while miR-296-3p is not, reflecting the complexity of ICAT modulation in GBM, which suggests that ICAT might be controlled by other mechanisms in grade II GBM [119].

Other possible signaling pathways targeted by miRNAs are the FOXG1 (Forkhead Box G1) and Smad6 pathways; FOXG1 is known to be an essential transcriptional factor in telencephalon development and was shown to be upregulated in multiple cancer cells, including gliomas [120]. One of the first studies connecting the dots between miRNAs and the FOXG1 pathway was carried out by Shibata et al. [121]. They suggested that the differentiation of neurons in the medial pallium of mice was regulated by miR-9 through FOXG1 signaling; more exactly, Zhen et al. suggested that miR-9-3p could be downmodulated in glioma cells, while the protein expression levels of FOXG1 were increased. They also identified FOXG1 as a direct target of miR-9-3p [122]. Furthermore, they observed suppressed proliferation and increased apoptosis of glioma cells transfected with miR-9-3p [122].

Likewise, miR-186 is significantly inhibited in tumor cells compared with adjacent nontumor tissues. Transfection of miR-186 into human glioma cells showed increased apoptosis through direct suppression of Smad6, which was negatively regulated by miR-186 levels [123]. Similarly, miR-142-3p also has antiproliferative functions and mitigates apoptosis of U87 and U251 glioma cells by blocking the transcription and translation of the high-mobility group box 1 (*HMBG1*) gene. By targeting *HMBG1*, miR-142-3p can inhibit the Wnt/β-catenin signaling pathway, and simultaneously initiates the caspase-3 pathway [124].

### miRNAs contributing to invasion and migration

Cancer progression begins with cell invasion, the migration of tumor cells from the primary origin to distant organs. Several miRNAs have been found to be valuable for the detection of the presence of metastases [125]. Recent studies using glioma cells have suggested that miR-21 is a key regulator of the expression of multiple genes and can thus induce several cellular programs in glioma cells, including invasion and microvascular proliferation of GBM [126]. miR-21 mediates these processes through inhibition of matrix remodeling genes, a reversion-inducing cysteine-rich protein with Kazal motifs [127], and tissue inhibitor of metalloproteinases 3 (TIMP3), which normally regulates the levels of matrix metalloproteinases (MMPs). MMPs are proteolytic enzymes that promote tumor cell invasion by disrupting the extracellular matrix [128]. It was found that miR-21 could negatively regulate the mRNA and protein levels of RECK and TIMP3 in glioblastoma cells [129]. Overexpression of miR-21 is also related to the upmodulated Sox2 (SRY-Box transcription factor 2), and Sox2 downmodulation can inhibit miR-21-enhanced glioma cell migration and invasion [130]. miR-21 expression levels are also associated with the grade of the glioma, being predominantly present at lower concentrations in grade II and III gliomas. GBM, in contrast, exhibits higher levels of miR-21, which is assumed to be correlated with its higher microvascular texture and ECM reorganization [24, 131]. In support of these findings, Gabriely et al. reported that vascular morphogenesis- and angiogenesis-related genes were significantly downregulated in response to miR-21 suppression in A172 glioma cells [24].

Matrix metalloproteinases (MMPs) are enzymes that catalyze the degradation of ECM and cell adhesion molecules (CADMs) that are essential in the cell-to-ECM attachment during the process of cell adhesion [132]. Interestingly, MMPs and CADMs have been identified as emerging targets for miRNAs; for instance, the aforementioned miR-21 could target MMP inhibitors such as RECK, enhancing the ECM degradation, and increasing the motility and cell invasion [24]. Also, miR-25 is upregulated in glioblastomas, where its expression levels are closely linked to the stage of the disease. miR-25 knockdown markedly decreases tumor cell migration and invasiveness through enhancing the expression of cell adhesion molecule 2 (CADM2). CADM2 is significantly underexpressed in glioma cells, a finding that confirms CADM2 as a promising target for miR-25 [133].

Another potential oncogenic miRNA is miR-720, which directly upmodulates the transcription of invasion- and migration-related genes based on a study performed by Liu et al. This was the first study to demonstrate  the inhibition of threonyl-tRNA synthetase like-2 (*TARSL2*) as a direct target of miR-720 [134]. Moreover, t-RNA synthases have previously been evaluated in immune disorders for their therapeutic roles [135]. Moreover, plasma lysyl-tRNA synthases have been shown to be associated with colorectal cancer [136, 137], enhancing cell adhesion for migration and metastasis [138]. In contrast, it seems that in GBM, threonyl-tRNA synthetase acts against miR-720 effects, promoting anti-invasive properties [134].

Conversely, tumor-suppressive miR-146b-5p has been proved to be negatively associated with its targeted gene, MMP16. The expression levels of miR-146b-5p in glioma cells are significantly low, while MMP16 show a surge in the same cells. Furthermore, the overexpression of miR-146b-5p was shown to promote degradation of MMP16-related mRNA. In this regard, the transfected U87 glioma cells presented shorter protrusions in response to miR-146b-5p mimics; also, direct blocking of MMP-16 using exogenous siRNA restored the miRNA activity against migration and invasion of glioma cells [139].

miR-379-5p has also been reported to be downmodulated in gliomas; the overexpression of this miRNA leads to the suppression of tumor cell viability, migration, invasion, and epithelial-to-mesenchymal transition (EMT). Notably, miR-379-5p exerts it effects by detoxifying microsomal glutathione transferase 1 (MGST1) [140]. High levels of glutathione transferase undesirably correlate with chemoresistance and poor survival, as these enzymes may bind to chemotherapy-induced cytotoxic agents, blocking their tumoricidal effects [141, 142]. Consistently, lower levels of MGST1 induced by miR-379-5p are linked to the improved survival, according to a study performed by Yang et al. [140].

Over recent years, HMGB1 has been discussed as a key mediator of proliferation and inhibition of apoptosis in tumor cells via the Wnt/β-catenin pathway. The suppression of this mediator by miR-665 is closely associated with the blockade of tumor cell migration and invasion in glioma patients. In line with this finding, lower levels of miR-665 in glioma cells correlate with developed grades and lower performance scales [143].

Among many other signaling cascades involved in cell proliferation, EMT, etc., the PI3K/Akt pathway is known to enable oncogenic signaling in multiple cancer cells, due to its effects on the aforementioned processes [144–147]. In this context, miR-3175 and miR-134 have been shown to affect cell proliferation, as well as EMT, in gliomas; the former miRNA activates the signaling of interest and leads to invasion, whereas the latter blocks the pathway, resulting in tumor cell apoptosis, which is a desirable result in cancerous conditions [148, 149].

Moreover, miR-296 has been revealed to be increased in primary cancerous endothelial cells, and is associated with cell invasion and multidrug resistance of glioma cells [150]. Finally, miR-320a has been reported to impede glioma cell invasion and migration by targeting aquaporin 4 (AQP4), which was recently defined as a strong regulator of cell invasion and migration in glioma subjects [151].

### miRNAs affecting angiogenesis

Angiogenesis, in brief, is the formation of a new vascular network from the preexisting vasculature. Angiogenesis is an intricate process involving the proliferation, migration, and differentiation of vascular endothelial cells (ECs) as a result of the complex involvement of proangiogenic and antiangiogenic factors [152]. As in other solid tumors, the growth of cells in glioma greatly relies on an adequate supply of oxygen and vital nutrients, which is principally provided through new blood vessels. Glioma angiogenesis is triggered by a variety of angiogenic factors and genes. More interestingly, miRNAs have been found to act as modulators of angiogenesis by activating and/or inhibiting the relevant molecular pathways. These types of miRNAs are named “angiomiRs,” playing significant roles in controlling the vascular network properties of gliomas [153].

An angiomiR characterized by being markedly downregulated in GBM-associated endothelial cells (ECs) is miR-125b, which targets *myc*-associated zinc finger protein [154]. Indeed, MAZ is a transcription factor that can regulate the transcriptional activation of vascular endothelial growth factor (VEGF) and VEGF receptors. Therefore, the overstimulation of miR-125b inhibits tumor angiogenesis [155, 156]. A hypoxic microenvironment typically surrounds glioma cells and results in the promotion of tumor angiogenesis through the regulation of particular miRNAs. In this setting, miR-210-3P is a hypoxia-regulated miRNA that is defined as a positive modulator of the activity of VEGF, carbonic anhydrase 9 (CA9), and HIF [90, 102].

Functional in vitro analysis has shown that miR-218 expression is considerably decreased in GBM compared to normal tissues; miR-218 indirectly targets HIF-2α through RTK signaling pathways. Thus, miR-218 downregulation could further influence GBM tumor neovascularization [157]. On the other hand, in vivo data suggest that miR-93 supports angiogenesis and EC activities via the induction of new blood vessel formation and the enhancement of EC survival through suppression of integrin-β8 [158].

Some other miRNAs, such as miR-124-3p, can control GBM cell proliferation, migration, and angiogenesis through activating the PI3K/Akt/NF-κB signaling pathway; it has been reported that the overexpression of miR-124-3p restricts glioma angiogenesis [152].

### miRNAs on the journey to drug resistance

Despite valuable advances in chemotherapeutic strategies, achieving successful therapeutic outcomes in glioblastoma patients has remained a major clinical hurdle, mostly owing to multiple resistance mechanisms [159]. It has been concluded on the basis of several studies that specific miRNAs involve in the chemoresistance of tumor cells through targeting drug-resistance-related genes or alterations in gene expression patterns in relation to distinct cellular processes, such as apoptosis, cell proliferation, and cell cycle modifications [160]. Hence, if such miRNAs are impaired and/or deregulated, they might act as deregulated gene modulators, leading to the drug resistance [161]. Depending on their specific target gene or mRNA, these miRNAs can possess cell specific activities, meaning that a single miRNA may have both tumor-suppressive and oncogenic potential [162–164].

Temozolomide (TMZ) has been widely accepted as the standard chemotherapeutic agent for GBM and astrocytoma. Since the introduction of TMZ as the first-line chemotherapeutic drug of choice for gliomas, high levels of drug resistance have occurred in at least half of all treated cases [165]. One of the accepted explanations is linked to miRNAs, as the expression profiles of several miRNAs have been shown to be upregulated in TMZ-resistant cells [160]. For instance, miR-548m is a downstream target of circular RNA-GLIS3 sponging activity, which upregulates MED31 mRNA expression, causing tumor cell invasion in TMZ-resistant cells [166].

The overexpression of miR-21 has been demonstrated to be linked to attenuated TMZ-induced apoptosis in human glioblastoma U87MG cells [167]. In particular, miR-21 mediates this by impairing the balance between proapoptotic Bax and antiapoptotic Bcl-2 proteins, through exerting a decrease in the Bax-to-Bcl-2 ratio, and also by decreasing the activity of caspase-3 [167, 168]. Similarly, miR-125b-2 confers GBM stem cells with resistance to TMZ; in TMZ-treated GBM stem cells, inhibition of miR-125b-2 activity using peptide nucleic acid (PNA) decreases the Bax-to-Bcl-2 ratio compared to treatment with TMZ alone, which further impedes apoptosis and confers drug resistance [169].

In human glioma cell lines with different p53 expression patterns, miR-221/222 contribute to a remarkable therapeutic resistance against TMZ by induction of the expression of Bax, cytochrome c, and caspase-3 [170]. On the other hand, miR-128 and miR-149 are also mediators of drug resistance; they both contribute to TMZ chemosensitivity by Rap-1B-mediated cytoskeletal remodeling [171]. In a similar pattern, miR-181a/b/c/d are also reported to sensitize tumor cells and minimize TMZ resistance through the same mechanism [172]. On the other hand, Chen et al. reported that miR-29a can also sensitize the response of glioma cells to TMZ through regulating the p53/MDM2 feedback loop [173]. Indeed, p53-induced expression of miR-29a can cause aberrant expression of MDM2 targeted by the corresponding miRNA, thereby affecting the activity of the p53-miR-29a-MDM2 loop [173].

Microarray analyses of miRNAs in GBM cell lines have shown that miR-10a, miR-195, and miR-455-3p involve in the acquisition of TMZ resistance. Although the suppression of miR-445-3p and miR-10a moderately influences the effectiveness of TMZ, miR-195 inhibition could effectively reverse the TMZ resistance [174].

Oncogenic miR-155-3p is reported to be overexpressed in gliomas, with the ability to induce cell growth in A172 and U87 cell lines, while its inhibition promotes sensitivity to TMZ by direct upregulation of Six1 at the translational level, thus inducing cell arrest at the same point at which TMZ acts on the G1/S phase [175]. Zhou et al. demonstrated that miR-141-3p increased cell growth and drug resistance through suppressing p53 and its downstream proteins such as cyclin-dependent kinase 2 (CDK2) and cyclin E1/B1, thus inhibiting cell cycle arrest at the G1 to S phases [176].

In contrast, miR-195 can decrease and reverse U251 glioma cells’ resistance by targeting oncogenic *CCNE1* [177]. CCNE1 (cyclin E1) is considered to be a potential subunit for CDK2, promoting cell progress from the G1 to S phase [178]. In this regard, miR-524-5p and miR-324-5p also inhibit U251 and U87 cell growth, and increase tumor cells’ chemosensitivity by downregulating the methyltransferase enhancer of zeste homolog 2 (*EZH2*) oncogene, which was previously identified to play a crucial role in TMZ resistance [179, 180]. Additionally, miR-524-5p has been confirmed to be an independent prognostic factor for improved survival [180].

In the case of TMZ resistance, miRNAs have also been indicated to be sponged during the process of resistance. In this setting, the upregulation of long- noncoding RNA MSC-AS1 in TMZ-resistant glioma cells has been linked to the PI3K/Akt oncogenic pathway, and its knockdown could profoundly suppress cell proliferation and drug resistance through sponging miR-373-3p. Cytoplasmic polyadenylation element-binding 4 (*CPEB4*), which encodes a cell survival protein, is a possible target gene for this miRNA [181]. In other words, MSC-AS1-induced inhibition of miR-373-3p causes *CPEB4* upmodulation, and therefore promotes cell growth and TMZ resistance through activation of the mentioned signaling pathway [182].

### miRNAs in self-renewal and differentiation of glioma stem cells (or cell stemness)

Recent studies support that miRNAs are critical regulators of stem-like features. miRNA profiles of glioma stem cells (GSCs) have been evaluated, revealing unique miRNA signatures in CD133^+^ GSC population compared to CD133^−^ non-stem-cell populations [183]. As described above, particular miRNAs could regulate major signaling pathways in the evolution of gliomas, including EGFR/RAS/NF1/PTEN/PI3K signaling and those related to p53, i.e. MDM2/MDM4/p14^ARF^ and p16^INK4a^/CD4/RB1 pathways. Furthermore, miRNAs are key molecular players in relation to biological characteristics of GSCs such as self-renewal and differentiation [184].

According to the roles of miRNAs in the differentiation and self-renewal of GSCs, they can be categorized into two subgroups: pluripotent miRNAs and prodifferentiation miRNAs. Pluripotent miRNAs are capable of increasing the self-renewal capacity and proliferation of stem cells but impede cell differentiation [185]. miR-18 and miR-137 are clear examples in this regard. Conversely, prodifferentiation miRNAs, including miR-9/9, miR-17, miR-34a, miR-124, miR-128, miR-137, miR-141, miR-145, miR-202, miR-326, miR-302–367 cluster, miR-451, and miR-504, can stimulate or stabilize differentiation of GSCs to more mature phenotypes [186]. As an example, in CD133^+^ A172 GBM cells transfected with miR-451 in combination with imatinib mesylate treatment, GSC growth and neurosphere formation were markedly inhibited [187]. miR-137 is another prodifferentiation miRNA with low expression levels in GSCs, caused by promoter hypermethylation. miR-137 exerts its effects by targeting RTVP-1, which itself suppresses the expression of CXCR4 [188]. C-X-C chemokine receptor type 4 (CXCR4) involves in several GSC characteristics, including generation and self-renewal. Moreover, miR-504 expression levels are significantly downregulated in GSCs compared to NSCs. However, transducing GSCs with miR-504 inhibits their self-renewal by targeting *Grb10*, which is considered to be an oncogene in GSCs and GBM [189].

Glioblastoma chemoresistance and recurrence are also considered to be the results of the activation and self-renewal of cancer stem cells [190, 191]. Researchers have shown that Nanog can negatively associate with AP-2α protein levels in human and mouse glioma cells and tissues [192]. As an aside, Nanog is a protein contributing to mutual crosstalk between GSCs and miRNAs, and is also linked to tumor aggressiveness, especially at high levels [193]. Nanog was identified to be the target of oncogenic miR-26a and was associated with the chemosensitivity of U251 cells. This oncogenic miRNA inhibits AP-2α expression, resulting in upmodulation of Nanog, and thus allowing for GSC renewal and drug resistance [192].

In contrast, miR-30a has anti-GSC activities, as it downregulates ecto-5′-nucleotidase (NT5E) levels, which further inhibits the Akt signaling pathway and ultimately decreases GSC clone formation, as well as  the proliferation in vitro and in vivo [194]. Investigation of rat-derived GSCs (C6 cells) has demonstrated that miR-30c overexpression can result in C6 cell sphere formation and neural differentiation to astrocytes through activation of the JAK–STAT signaling pathway [195]. miR-33a acts as another oncomiR, which is upregulated in GSCs and directly increases phosphodiesterase 8A (PDE8A) and ultraviolet (UV) radiation resistance-associated gene/protein (*UVRAG*) mRNAs’ expression; the encoded proteins have been reported to be responsible for regulating protein kinase A (PKA) and Notch endocytosis signaling pathways, respectively [196].

miR-300, which is extremely prevalent in glioma cells and tissues, and exists in even higher amounts in GSCs [197], increases cell proliferation and self-renewal-related activities in patient-derived GSCs; however, it inhibits stem cell differentiation, maintaining the GSCs’ undifferentiated status. Interestingly, it seems that all these effects are mediated by direct suppression of leucine zipper putative tumor suppressor 2 (*LZTS2*) [197].

## miRNAs as promising diagnostic/prognostic biomarkers for gliomas

Early diagnosis of glioblastoma can remarkably reduce mortality and improve patient outcomes and quality of life. However, identifying early-stage glioblastoma is still very complicated. Molecular prognostic and diagnostic biomarkers have been introduced recently by the World Health Organization (WHO) into the classification of CNS tumors. Recognition of these biomarkers in tissues and/or body fluids is crucial to characterizing subclasses of tumors that cannot be precisely classified and/or cannot be analyzed due to scant tissue samples [198].

Several studies have shown a correlation between the expression patterns of specific miRNAs and the development/growth of glioblastomas. Moreover, circulating miRNAs could serve as potential cancer biomarkers through exosome-mediated intercellular communication. Exosomes containing miRNAs are released either from viable cells or apoptotic bodies, and can circulate through serum and/or cerebrospinal fluid (CSF) [199]. Therefore, measurement of these circulating miRNAs may provide reliable, accessible diagnostic tools for multiple malignancies, including gliomas. To date, tremendous effort has been made to establish a miRNA panel for glioma models with capability for diagnostic and prognostic purposes [199, 200]. A recent study in this regard reported that the expression levels of miR-21, miR-222, and miR-124-3p are increased in serum exosomes derived from patients with aggressive high-grade glioblastoma, suggesting that these miRNAs may serve as biomarkers for predicting tumor progression at early stages [201].

Another study demonstrated that coexpression of miR-15b and miR-21 could be a valuable diagnostic tool in gliomas. Additionally, it has been reported that miR-16 could be exploited to discriminate glioblastoma from other types of glioma [202].

A specific analysis of Cancer Genome Atlas data revealed an inverse connection between miR-196b/miR-10b levels and overall survival of glioblastoma patients [203]. Another prognostic miRNA in this field is miR-328, whose low expression levels correlated with poor patient survival [204]. Similarly, the expression levels of miR-549a and miR-502-5p have prognostic significance in patients with tumors of glial origin [205]. Some miRNAs could also be used for monitoring tumor progression. In this context, studies evaluating miR-205 in glioma patients have indicated that following surgery, miR-205 content is increased, whereas its serum concentrations decrease after the recurrence period [206].

The implications of using miRNAs as diagnostic and predictive biomarkers for glioblastoma have been reviewed comprehensively [207]; nevertheless, few assessments have evaluated their prognostic potential in lower-grade gliomas [208]. Meanwhile, individual prognostic miRNAs have been investigated in multiple studies; for instance, a meta-analysis suggested that the upmodulation of miR-15b, miR-21, miR-148a, miR-196, miR-210, and miR-221 and downregulation of miR-106a and 124 are consistent with poor survival in glioma patients [209].

However, there are controversial results regarding prognostic miRNA signatures in literature, which could be due to small sample size, shorter follow-up times, and the use of different assays and normalization techniques. This is more obvious in lower-grade gliomas, as these entities are less common and are often lost to follow-up owing to longer survival times. A recent analysis evaluating a 30-miRNA prognostic model suggested that glioblastomas (*n* = 35) can be classified into two subgroups with early death (< 450 days) versus long-term survival (> 450 days) [210]. A more comprehensive analysis using a larger patient population (*n* = 563) from the TCGA cohort identified three miRNAs (incl. miR-222, miR-302d, and miR-646) that independently acted as survival biomarkers [211]. Using a TCGA cohort, Hayes et al. also offered a risk assessment score according to nine miRNAs that were significantly associated with survival [212]. Surprisingly, miR-222 was found to be a common contributor in all three studies; however, it was not confirmed to be a disease-free survival biomarker in another study that used the same dataset [213]. These inconsistencies raise the need for not only larger patient cohorts but also adjustment for many confounders such as the grade and histology of gliomas [214].

Hayes et al. identified eight miRNAs (incl. miR-124a, miR-202, miR-7, miR-222, miR-363, miR-630, miR-663, and miR-204) with the ability to predict overall survival in those treated with bevacizumab [207]. Interestingly, miR-7, an antiangiogenic miRNA discussed above, has been reported to correlate with poor response to bevacizumab, suggesting that tumors not enriched with vascular networks will not exhibit an ideal response to this VEGF-targeting chemotherapeutic agent. Additional assessments have also investigated individual miRNAs and miRNA profiles for predicting responsiveness to TMZ, in addition to radiation therapy [215] or alone [216]; however, it remains to be concluded whether their predictive potentials are generalizable to clinical settings or not.

## Therapeutic potential of miRNAs

miRNAs simultaneously regulate multiple genes and proteins across different signaling pathways. This regulatory characteristic makes miRNAs promising therapeutic targets for multiple diseases; therapeutics act as either miRNA mimics or miRNA inhibitors. miRNA inhibitors are single-stranded oligonucleotide antagonists [217, 218], while anticarcinogenic miRNAs could be induced through double-stranded miRNAs, formed synthetically according to the biological miRNA twin sequence. Accordingly, different aspects of miRNAs’ therapeutic potential in gliomas are discussed in this section (Fig. 1).

The first miRNA-based therapeutic approach is to inhibit the action of miRNAs; miRNA antagonists (antagomiRs) usually bind to oncomiRs and inhibit their action by blocking miRNA-to-mRNA attachment, a process which is called antisense action. Indeed, antagomiRs are specific and irreversible miRNA antagonists, responsible for further degradation of miRNAs [219]. Conversely, miRNA masks bind to mRNAs and mask the target 3′-UTR sequence, preventing the miRNA from acting on mRNA. Therefore, miRNA masks do not degrade miRNAs and allow their off-target actions on other genes to be left intact [220]. Another mechanism for inhibiting a miRNA is obtained through sponging of miRNAs [221, 222]. A sequence of RNA complementary to the binding site of a particular miRNA, or even a set of miRNAs with similar seed sequences, occupies this binding site and prevents all biological miRNAs from acting on mRNAs. Due to the fact that these sponges are not chemically modified for specific miRNAs, they may have low affinity and require higher concentrations to act as a proper inhibitor. Additionally, there is a requirement for strong promoters and the necessity for multiple vector integration [222, 223].

When using the second mechanism, which is defined as induction of the activities of miRNAs, anticarcinogenic miRNAs could induce tumor-suppressive activities by miRNA mimics, through reversing epigenetic silencing. Small-molecule modulators are considered to be excellent cancer therapeutics, based on their high stability [224]. miRNA silencing in carcinogenic signaling pathways is reversed by hypomethylating agents such as 5-azacytidine [225]. Interestingly, these agents can reexpress multiple mRNAs, miRNAs, as well as the other noncoding RNAs. Synthetic RNAs acting as native miRNA twins include one RNA strand (the guide strand) that is identical to the target miRNA and act by “mimicking” the functions of the mature miRNAs. The passenger strand may be either completely or partially compatible with the guide strand [226, 227]. Any type of incompatibility or side-compatibility with other miRNAs could result in side effects; therefore, the design process should be performed carefully and monitored before introduction into viable tumor cells [228].

In the case of the third therapeutic strategy, i.e., monitoring the delivery systems, a precise evaluation of the delivery of therapeutic miRNAs is essential, since many potential side effects may occur during in vivo systemic administration. For instance, chemically modified miRNAs are crucial for conserving their viability and stability while passing through the bloodstream. These alterations actually protect miRNAs from nuclease-induced degradation, as well as their renal clearance or reticuloendothelial removal [229–231]. Furthermore, researchers have also suggested the use of viral vectors for this purpose, in which regard careful consideration of immune system responses is definitely required. Accordingly, most approaches prefer nonviral vectors for successful delivery of miRNA therapeutics [218, 232]. Nanocarriers can also protect oligonucleotides, such as miRNA mimics or antioligomers, from sequestration by nucleases. Nanoparticles made of Gold (Au) or other inorganic materials such as silicon/iron oxide are considered to be safe carriers for oligonucleotides. Au, as an inert element, has been shown to cross the blood–brain barrier (BBB), which could make it particularly suitable for glioma patients [233, 234]. Other nanoparticles, such as polyethylene glycol [235]–polyethylenimine (PEI) liposomes, have been reported to exhibit longer viability and lower immunogenicity, and thus could be employed to treat various cancers. However, minimal changes in their cationic head or hydrophobic chain may result in dramatic immune responses or hinder their transfection [234, 236].

Despite all the developments in miRNA-based therapeutic strategies, designing appropriate delivery systems for patients with glioblastoma remains challenging due to the presence of the BBB. However, cell-penetrating peptides and immunoliposomes have been shown to be promising in terms of crossing the BBB [237]. Finally, after introducing miRNAs into tumor cells, there are many concerns regarding the targeting of specific actions or side effects, and many other biological barriers such as high interstitial tumor pressure or alterations in ECM may affect the final outcome [238].

## Conclusions

The modulatory significance of miRNAs in glioma pathogenesis and progression is no longer unknown. The role of these ncRNAs in different glioma-related cellular and molecular processes, such as cell proliferation and apoptosis, migration and invasion, angiogenesis, drug resistance, and cell stemness and differentiation, as discussed comprehensively in this review, has been revealed. Based on this suggested correlation, any type of disturbance in the miRNA modulatory plexus could influence the pathogenesis of glioma. Since various human miRNAs have been identified to date, it is worth precisely profiling miRNAs that are highly expressed in glioma cell and tissue specimens. Moreover, establishing molecular targets, as well as relevant cellular pathways, is required to define the miRNA–glioma relationship. On the other hand, miRNAs have also provided novel capacities in the clinical setting; they can potentially act as glioma biomarkers for both diagnostic and prognostic purposes. Additionally, miRNA-based therapeutic approaches are very promising for the management of gliomas. The introduction of newly identified systems for the delivery of miRNAs and their transfer across the BBB heralds a bright future for glioma and GBM therapy. Notwithstanding, further investigations, especially in large cohorts at different laboratories and research centers, are still needed to further clarify the diagnostic and therapeutic potential of miRNAs in relation to gliomas and glioma patients.

## Data Availability

Not applicable.

## Abbreviations

CADM2: Cell adhesion molecule 2
CDK2: Cyclin-dependent kinase 2
ECM: Extracellular matrix
EGF: Epidermal growth factor
EMT: Epithelial-to-mesenchymal transition
GBM: Glioblastoma multiforme
HGF: Hepatocyte growth factor
IDH: Isocitrate dehydrogenase
MGST1: Microsomal glutathione transferase 1
miRNAs: MicroRNAs
MMPs: Matrix metalloproteinases
NcRNAs: Noncoding RNAs
PDGF: Platelet-derived growth factor
PiRNAs: Piwi-interacting RNAs
RISC: RNA-induced silencing complex
TMZ: Temozolomide
TNFRs: TNF-α receptors
TRBP: TAR RNA-binding protein
VEGF: Vascular endothelial growth factor
